# Placental-fetal distribution of carbon particles in a pregnant rabbit model after repeated exposure to diluted diesel engine exhaust

**DOI:** 10.1186/s12989-023-00531-z

**Published:** 2023-05-18

**Authors:** Eva Bongaerts, Tim S Nawrot, Congrong Wang, Marcel Ameloot, Hannelore Bové, Maarten BJ Roeffaers, Pascale Chavatte-Palmer, Anne Couturier-Tarrade, Flemming R Cassee

**Affiliations:** 1 Centre for Environmental Sciences, Hasselt University, Agoralaan Building D, 3590, Diepenbeek, Belgium; 2 Department of Public Health and Primary Care, KU Leuven, Kapucijnenvoer 35 blok d-box 7001, Leuven, 3000 Belgium; 3 Biomedical Research Institute, Hasselt University, Agoralaan Building C, Diepenbeek, 3590 Belgium; 4 Department of Microbial and Molecular Systems, KU Leuven, Celestijnenlaan, Leuven, 200F-box 2454, 3001 Belgium; 5 Université Paris-Saclay, UVSQ, INRAE, BREED, Jouy-en-Josas, 78350 France; 6 Ecole Nationale Vétérinaire d’Alfort, BREED, Misons-Alfort, 94700 France; 7 National Institute for Public Health and the Environment, RIVM, PObox1, Bilthoven, 3720 BA the Netherlands; 8 Institute for Risk Assessment Sciences, Division Toxicology, Utrecht University, Utrecht, the Netherlands

**Keywords:** Airborne pollution, Diesel exhaust, Gestational exposure, Label-free detection

## Abstract

**Background:**

Airborne pollution particles have been shown to translocate from the mother’s lung to the fetal circulation, but their distribution and internal placental-fetal tissue load remain poorly explored. Here, we investigated the placental-fetal load and distribution of diesel engine exhaust particles during gestation under controlled exposure conditions using a pregnant rabbit model. Pregnant dams were exposed by nose-only inhalation to either clean air (controls) or diluted and filtered diesel engine exhaust (1 mg/m^3^) for 2 h/day, 5 days/week, from gestational day (GD) 3 to GD27. At GD28, placental and fetal tissues (i.e., heart, kidney, liver, lung and gonads) were collected for biometry and to study the presence of carbon particles (CPs) using white light generation by carbonaceous particles under femtosecond pulsed laser illumination.

**Results:**

CPs were detected in the placenta, fetal heart, kidney, liver, lung and gonads in significantly higher amounts in exposed rabbits compared with controls. Through multiple factor analysis, we were able to discriminate the diesel engine exposed pregnant rabbits from the control group taking all variables related to fetoplacental biometry and CP load into consideration. Our findings did not reveal a sex effect, yet a potential interaction effect might be present between exposure and fetal sex.

**Conclusions:**

The results confirmed the translocation of maternally inhaled CPs from diesel engine exhaust to the placenta which could be detected in fetal organs during late-stage pregnancy. The exposed can be clearly discriminated from the control group with respect to fetoplacental biometry and CP load. The differential particle load in the fetal organs may contribute to the effects on fetoplacental biometry and to the malprogramming of the fetal phenotype with long-term effects later in life.

**Supplementary Information:**

The online version contains supplementary material available at 10.1186/s12989-023-00531-z.

## Introduction

Air pollution has been estimated to cause 6.7 million premature deaths worldwide in 2019 [1]. The associated burden of disease is linked to air pollution affecting the cardiovascular, respiratory and neurological systems [2–4]. Accordingly, air pollution is recognised as the biggest environmental threat to human health [1]. Traffic-related air particulate pollution constitutes a substantial portion of outdoor air pollution globally, with a significant contribution by diesel engines used in vehicles such as cars, trains and boats. Diesel engine exhaust is a complex mixture of hundreds of constituents in either gas or particle form. Gaseous components of diesel exhaust include carbon monoxide, oxygen, nitrogen, water vapour, nitrogen compounds, sulphur compounds and numerous low-molecular-weight hydrocarbons (some of them individually known to be toxic, such as aldehydes, benzene, 1,3-butadiene, polycyclic aromatic hydrocarbons (PAHs) and nitro-PAHs). The particles present in diesel exhaust are known to be composed of a centre core of elemental carbon with absorbed organic compounds and small amounts of sulfate, nitrate, metals and other trace elements [5, 6]. In addition to the effects observed in adult life, a growing body of evidence supports a link between prenatal particulate matter (PM) exposure and adverse pregnancy and birth outcomes in both animal [7, 8] and population-based studies [9–11]. CPs present in diesel engine exhaust could disturb fetal development via maternal exposure by (i) deregulating the placental function, for example, through changes in the intra-placental blood flow [8, 12, 13] or (ii) generating inflammation and oxidative stress in the placenta [14–16]. In this regard, prenatal exposure to fine particles from diesel engine exhaust has been shown to increase the risk of having children with intra-uterine growth retardation [10] or small for gestational age [11, 17]. Recent work on the presence of CPs from ambient exposure in human fetal tissues [18] gives further cause for concern on maternal inhalation of PM during pregnancy and their distribution inside the intrauterine developing fetus. Moreover, work on the biodistribution of inhaled nanomaterials also suggests that such small particles can reach and affect the placenta and fetal tissues [19–22].

In this study, using a previously developed rabbit model [8], we employ controlled nose-only exposure to diluted diesel engine exhaust throughout gestation to simulate environmental exposures to combustion-derived particulate matter during pregnancy. Our aim was to study the placental-fetal distribution and to quantify the internal fetal particle loads to confirm transplacental CPs after controlled *in utero* exposure to diluted diesel engine exhaust. Rabbits were used as models because of their haemodichorial placentation, which is anatomically and functionally closer to that in humans than that in rodents. Moreover, controlled nose-only exposure was used as it is more relevant to human exposure than the more often used whole body exposure, which does not discriminate effects due to inhalation from those due to ingestion after self-grooming. Based on this controlled nose-only inhalation study, previous studies have already demonstrated disturbance of the fetoplacental development [8] and an increased risk of developing cardiometabolic disorders [23] following maternal exposure to diesel engine exhaust. Translocation of CP-like structures (most probably originating from the diesel exhaust) from the dams to the fetal systemic circulation across the placenta has been proposed as a possible underlying mechanism for developing these adverse health effects. Here, we show the transfer of CPs from diesel exhaust towards the fetal circulation and organs using white-light generation by CPs under ultrashort pulsed laser illumination as a sensitive detection technique.

## Materials and methods

### Animal exposure

The animal exposure details have been described by Valentino et al. 2016 [8]. Fourteen pregnant New Zealand white female rabbits (INRA1077 line, 1-year old) were exposed by nose-only inhalation in custom-made Plexiglas tubes to either diluted diesel engine exhaust (1 mg/m^3^) (exposed group; N = 7) or clean purified air (control group; N = 7) for 2 h/day (hence equivalent to a 24 h average of ~ 85 *µ*g/m^3^), 5 days/week, from the 3rd to the 27th day post-conception (i.e., 20 days altogether over a 31-day gestation). Inhalation exposure was performed for 1 h in the morning and 1 h in the afternoon to mimic the daily commuting between home and work. The rest of the time, the rabbits were housed in their individual cage in a temperature, light cycle, hygrometry and air renewal-controlled atmosphere. Animals had *ad libitum* access to water and food. The experimental procedure for animal exposure has been approved by the French ethical committee N°45 under the number N°12/102. Diesel engine exhaust exposure was performed by restraining the rabbits in dedicated nose-only tubes inside an exposure chamber that was connected to a 25KVA Loxam diesel engine (Loxam, Ridderkerk, The Netherlands) [24]. The measured components of the diesel exhaust exposure mixture used in the present experiment were analysed elsewhere [8] and shown in **Supplementary Table 1** (Additional file 4), and their nanoparticle content displayed an average median size of 69 nm.

### Tissue collection

After euthanasia on GD28, fetoplacental units (from 32 control and 32 exposed fetuses) were collected. Fetuses and placentas were weighed prior to dissection. Fetuses were sexed by visual observation of the internal genital organs. Pieces of labyrinthine area and fetal organs (i.e., heart, kidney, lung, liver and gonad) were fixed with formalin. Samples were dehydrated in xylene, embedded in paraffin and then cut into 7 *µ*m thick sections using a microtome (Leica Microsystems, UK), floated onto charged glass slides (Super-Frost Plus, Fisher Scientific, USA) and dried overnight at 37 °C.

### Carbon particle detection

**Fig. 1:**
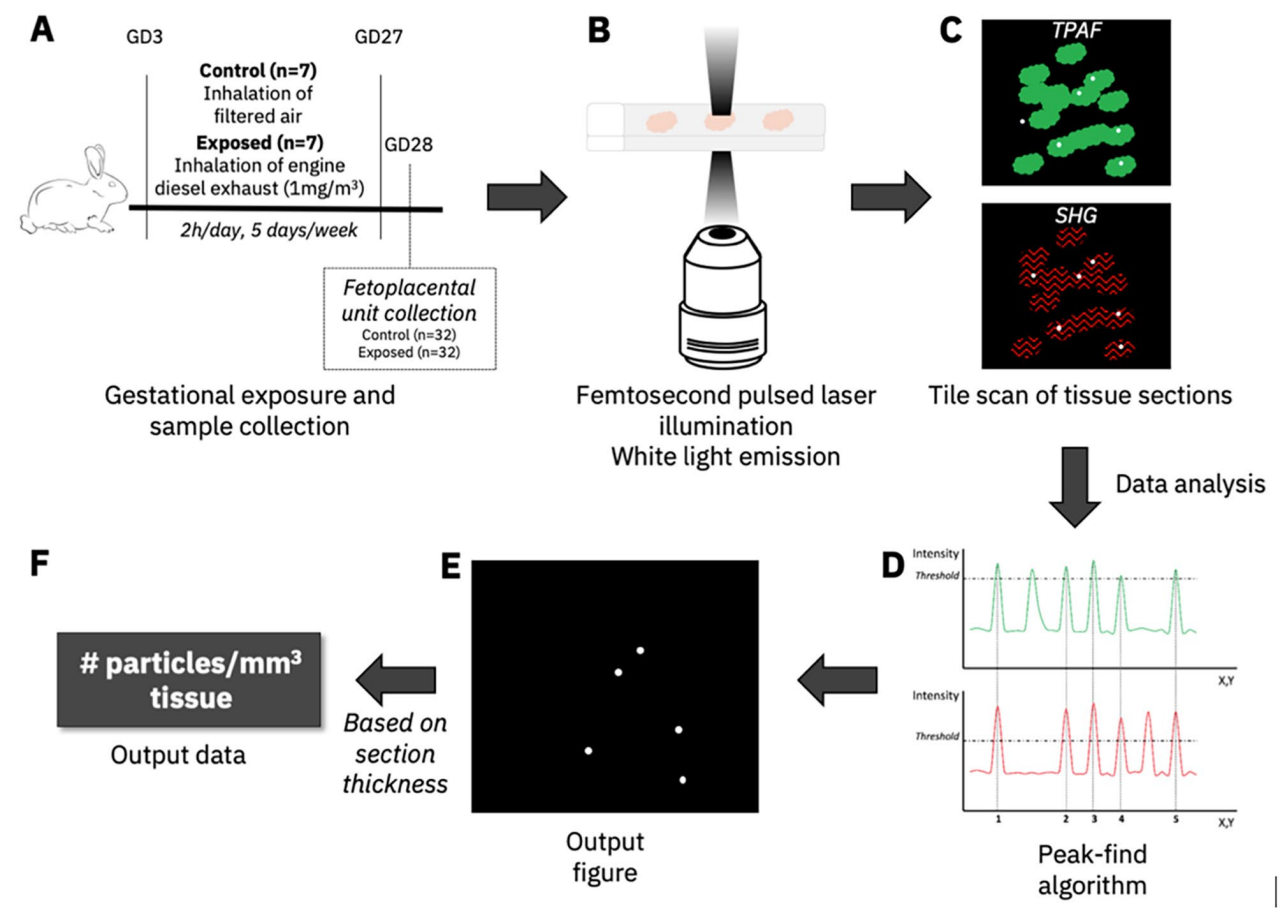
**Experimental protocol for carbon particle detection in rabbit fetoplacental units.** (A) Pregnant New Zealand white rabbits (INRA1077 line, 1-year old) were exposed by nose-only inhalation in custom-made Plexiglas tubes to either clean purified air (control group, n = 7) or diluted diesel engine exhaust (1 mg/m3, exposed group, n = 7) for 2 h/day, 5 days/week from GD3 to GD27. After euthanasia on GD28, fetoplacental units (from 32 control and 32 exposed fetuses) were collected. Placenta, fetal heart, kidney, lung, liver and gonads were formalin-fixed, paraffin embedded, and sections of 7 *µ*m were prepared. (B) The tissue sections are illuminated using a two-photon femtosecond pulsed laser tuned to a central wavelength of 810 nm. (C) The white light signal generated by the CPs present in the tissue (white dots) is simultaneously detected with two-photon excited autofluorescence (TPAF) of the tissue cells (green) and second harmonic generation (SHG) from collagen (red). (D) The number of CPs in the obtained images is determined using a peak-finding algorithm which counts connected and thresholded pixels. (E) CPs (white dots) in the output figure were defined as the thresholded pixels found in both detection channels. (F) Finally, based on the tissue thickness, the results are expressed as the number of detected CPs per cubic millimetre of tissue. Abbreviations – CP: carbon particle; GD: gestational day

The fetoplacental CP load, associated with inhaled diluted diesel engine exhaust, was measured in fetal heart, kidney, liver, lung, gonad and placenta from gestationally exposed and control pregnant dams, using non-incandescence related white-light generation under femtosecond pulsed laser illumination [20, 21]. As depicted in Fig. 1, the present CPs were analysed based on two of the characteristic white-light features: (i) the emission signals saturate compared with other label-free signals (e.g., second harmonic generation and two-photon excited autofluorescence), which allows thresholding of the particles in the detection channels and (ii) the emitted white light ranges over the whole visible spectrum; hence the CPs are detected by pixels thresholded in both channels simultaneously. Images from the fetoplacental tissue sections were collected at room temperature using a Zeiss LSM880 (Carl Zeiss, Germany) equipped with a femtosecond pulsed laser (810 nm, 150 fs, 80 MHz, MaiTai DeepSee, Spectra-Physics, USA) tuned to a central wavelength of 810 nm using an EC Plan-Neofluar 10x/0.30 M27 objective (Carl Zeiss, Germany). Two-photon induced white light emission of CPs was acquired in the non-descanned mode after spectral separation and emission filtering using 405/10 nm and 550/200 nm band-pass filters. The fetoplacental tissue sections were imaged using tiles scans. The sizes of the recorded tile scans were based on the covering tissue area and were recorded with a 4096 × 4096 pixel resolution, 0.83 *µ*m pixel size and 2.05 *µ*s pixel dwell time. A minimum of five tissue regions were imaged per sample. The images were acquired by ZEN Black 2.0 software (Carl Zeiss, Germany).

### Carbon particle quantification

To count the number of CPs in the recorded tile scans, an automated and customised MATLAB program (MATLAB 2010, MathWorks, The Netherlands) was used [25, 26]. First, a peak-finding algorithm detects connected pixels above a certain threshold value in both, namely 99.5% and 55%, from the highest pixel intensity of the narrow second harmonic generation channel (405/10) and two-photon excited autofluorescence channel (550/200), respectively. Next, the detected pixels in both channels are compared, and only the overlapping ones are used to generate the output and metrics. The average amount of CPs detected in the tissue tile scans was normalised for the tissue area determined using Fiji (ImageJ v2.0, Open source software, http://fiji.sc/Fiji). Finally, the results were expressed as the number of detected CPs per mm^3^ tissue by considering the tissue section thickness, namely 7 *µ*m.

In addition, optical sectioning in the z-direction throughout the placental and fetal tissue was performed to confirm tissue embedment and, with this, exclude external contamination. Approximately 48 images with a 1024 × 1024 pixel resolution and 0.21 *µ*m pixel size were acquired throughout the tissue sections with an optical slice thickness of 0.63 *µ*m using a pixel dwell time of 2.05 *µ*s. Orthogonal xz- and yz-projections were made using Fiji (ImageJ v2.0, Open source software, http://fiji.sc/Fiji).

### Data processing and statistical analysis

To assess the intervariability (i.e., the variability between the same organ from different fetuses within the same dam/litter) in CP load, we performed the parametric Kruskal-Wallis test with Dunn’s correction for multiple testing. The data were pooled per dam as no significant differences could be determined within the same litter. Accordingly, all statistics were performed on the average CP load per litter for the different tissues (n = 7 dams per group). Next, to evaluate the intravariability (i.e., the variability between different fetal organs from the same dam), we assessed the non-parametric Spearman correlation and Wilcoxon matched-pairs signed rank test to assess differences in the CP load in the different fetal organs. In addition, to compare the fetal CP load between control and exposed animals, the non-parametric Mann-Whitney test was used. Data are expressed as median (Q1; Q3) with first (Q1) and third (Q3) quartiles.

To further assess the association between organ weight or weight ratio and CP load in each fetal organ with adjustment for treatment and fetal sex, linear mixed-effects models were fitted using the R package nlme (version 3.1–153) with random intercept effect of dam (nlme package, R, Phinheiro, Bates, DebRoy, Sarkar and the R Development Core Team 2013. nlme. R package version 3.1–111;www.r-project.org/). For the placental CP load, a random slope effect was added in addition to a random intercept effect. CP load was log_10_-transformed, and the model estimates were expressed as the change in organ weight or weight ratio per 10-times increase in CP load. In order to have a global view of the biometrical analysis on the placental and fetal CP load, multiple factor analyses (MFAs) were additionally performed using the R package FactoMineR (version 2.4) [27]. MFA is a multivariate data analysis method for summarising and visualising a complex data table in which individuals are described by several sets of variables (quantitative and/or qualitative) structured into groups, where the variables are of the same nature in each group. MFA is thus a factorial method that can be used to reduce the dimension of data to assist in describing or subtyping samples. In MFA, quantitative and/or qualitative variables are grouped based on the nature of the variables. It can be seen as an extension of principle component analysis (PCA, with only quantitative variables) and multiple correspondence analysis (MCA, with only qualitative variables), balancing the grouping structure of the involved variables. A first MFA was performed with all variables and exposure, where variables were grouped into CP load, organ weight and weight ratio. Next, another MFA was performed with fetal sex additionally added. The first two principal components (PCs) were extracted for data visualisation of both the individuals and the variables. The individual’s factor map was shown with grouping information on exposure or exposure/sex, where 95% confidence ellipses around the group mean were added. In addition, a cos^2^ value far from 1 and a v.test > 1.96 indicated good separation. The variables factor map presents a view of the projection of the observed variables projected into the plane spanned by the first two PCs. The closer a variable is to the circle of correlations, the better its representation on the factor map (and the more important it is to interpret these components).

## Results

### Placental and fetal tissue carbon particle distribution

The fetoplacental CP load, associated with inhaled diluted diesel engine exhaust, was measured in fetal heart, kidney, liver, lung, gonad and placenta from gestationally exposed and control pregnant dams, using white-light generation under femtosecond pulsed laser illumination (Fig. 1) [25, 26].

**Fig. 2:**
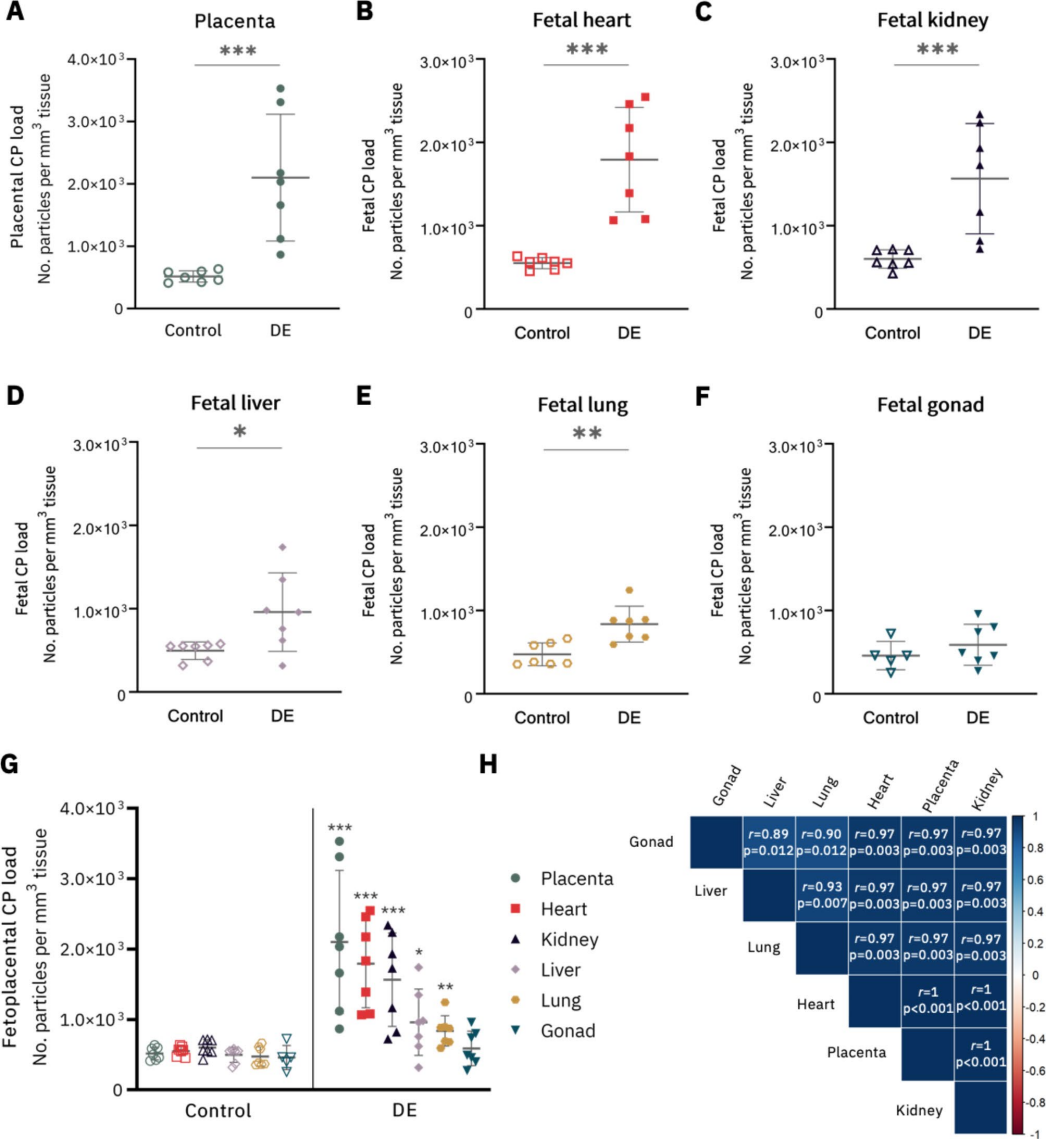
**Carbon particle load in placental and fetal tissues.** (A-G) CPs were present in all screened samples, and (H) a strong positive association in particle load was found between the fetal tissues within the exposed group as visualised by the heatmap and given by the corresponding two-sided Spearman correlation values. The empty and filled symbols are indicative of the individual fetal particle load in the control and exposed group, respectively. (A) The green dots represent the placental particle load, (B) the red squares the heart particle load, (C) the dark blue triangles the kidney particle load, (D) the purple diamonds the liver particle load, (E) the gold hexagons the lung particle load and (F) the light blue triangles the gonad particle load. (G) Differences in particle load between the control and exposed group were analysed by the Mann-Whitney test. (H) In addition, we used the non-parametric Spearman correlation and Wilcoxon matched-pairs signed rank test to assess the differences in particle load in the different fetal organs. The average particle load per litter (n = 7 dams per group) was shown and used for all analyses. *p ≤ 0.05, **p ≤ 0.01, ***p ≤ 0.001. Abbreviations – CP: carbon particle; DE: diesel exhaust

In the diesel engine exhaust exposure group, all organs, except the gonads, had significantly more CPs detected compared with the filtered air control dams as evaluated by the Mann-Whitney test (Fig. 2A-G). The particle load in the diesel engine exhaust exposed group was, on average, 4.1-fold higher in the placenta (p < 0.01), 3.3-fold higher in the fetal heart (p < 0.01), 2.6-fold higher in the fetal kidney (p < 0.01), 1.9-fold higher in the fetal liver (p < 0.05) and 1.8-fold higher in the fetal lung (p < 0.01) compared with the particle load in the corresponding organ from the control group. In addition, we observed high compartmental particle load correlations between the placental and fetal organs for the exposed group (Fig. 2H), indicating that higher placental particle loads were associated with an increased number of detected CPs in the corresponding fetal organs within one litter.

Compared with the placental tissue particle load, the accumulation of CPs was, on average, 15% lower in the fetal heart (p = 0.078), 25% lower in the fetal kidney (p < 0.05), 55% lower in the fetal liver (p < 0.05), 61% lower in the fetal lung (p < 0.05) and 72% lower in the fetal gonads (p < 0.05).

**Fig. 3:**
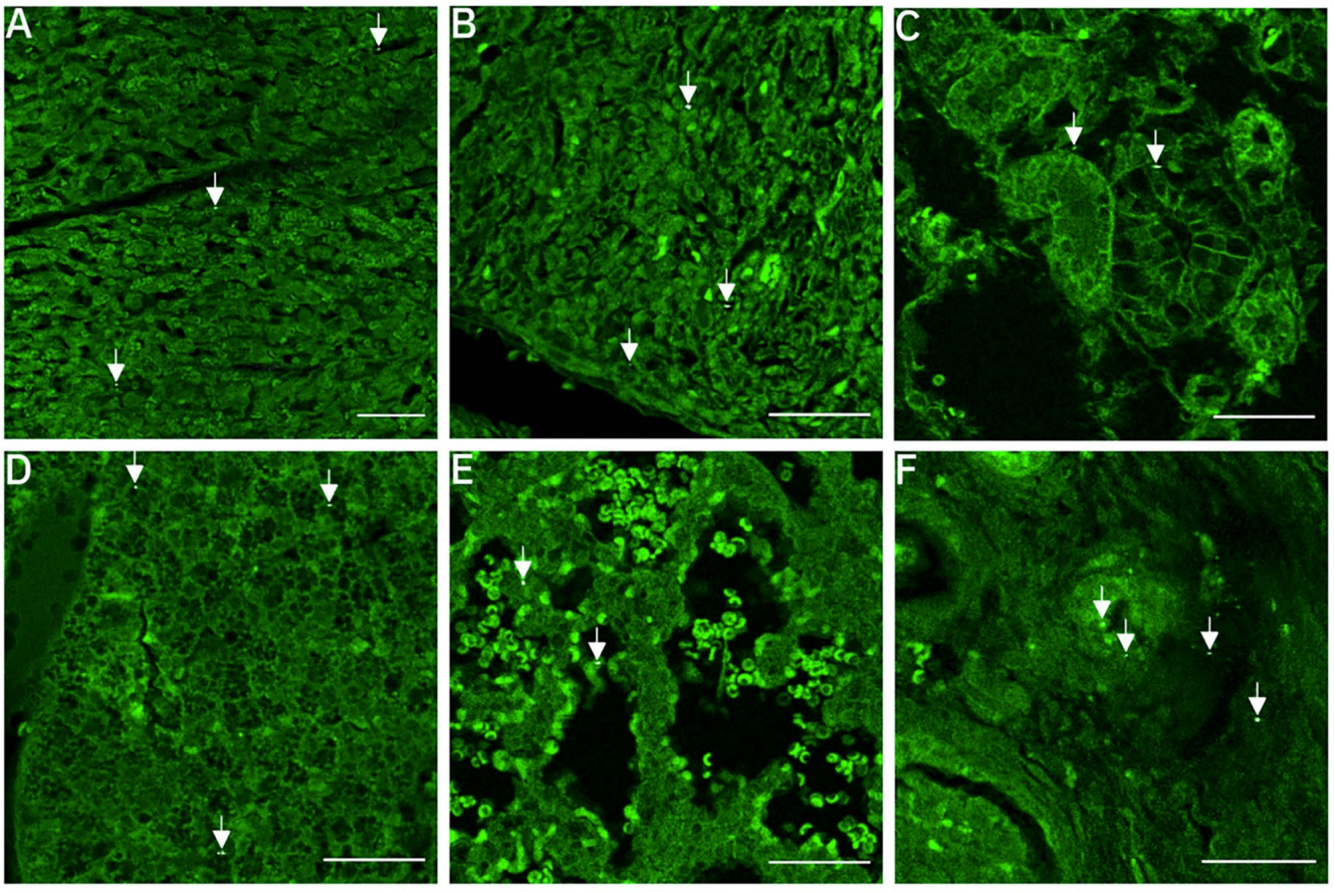
**– Placental-fetal distribution of carbon particles following gestational diesel engine exposure.** CPs were present (white and further indicated with white arrowhead) in the (**A**) placenta, fetal (**B**) heart, (**C**) kidney, (**D**) liver, (**E**) lung and (**F**) gonads. Magnification x20. Scale bars: 50 *µ*m. Abbreviations – CP: carbon particle

CPs were identified in all fetoplacental samples (Fig. 3A-F, **Supplementary Fig. 1** (Additional file 1)) and their embedment in the fetal and placental tissues excludes external contamination (**Supplementary Fig. 2** (Additional file 2)). CPs were located in the maternal blood space of the labyrinthine area in the lumen but also near the trophoblastic layer (Fig. 3A, **Supplementary Fig. 3** (Additional file 3)). Moreover, CPs were located in the cardiac tissue of the fetal heart (Fig. 3B**)**, in the lumen and cells of the urinary tubules of the fetal kidneys (Fig. 3C), fetal liver (Fig. 3D) and fetal lung (Fig. 3E). Lastly, CPs were detected in the fetal ovaries (Fig. 3F) and testes (not shown).

### Fetoplacental biometry

Using a previously developed rabbit model [8], we study the influence of gestational exposure to diesel engine exhaust on fetoplacental biometry. At GD28 (i.e., 3 days before birth), the placenta to fetus weight ratio (i.e., grams of placenta per grams of fetus) showed a positive trend in function of placental CP load (Table 1). A 10-times increase in placental CP load corresponds with a 0.046 (95% CI: -0.0021 to 0.095) increase in placenta to fetus weight ratio (p = 0.067). Other biometric parameters (i.e., fetal weight, organ weight), as well as fetal organ (i.e., heart, kidney, liver, lung and gonad) to fetal weight ratios, did not vary significantly with the CP load in the placental-fetal organs.

**Table 1:** **– Fetoplacental biometry at GD28.** Female pregnant rabbits inhaled either 1 mg/m^3^ of diesel exhaust (DE) or clean air (C) for 2 h/day, 5 days/week from GD3 to 27. Dams were euthanised, and fetoplacental units of control (C) and exposed (DE) groups were collected at GD28. The effect of gestational exposure to diesel engine exhaust on the fetoplacental biometry was estimated by assessing the association between organ weight or weight ratio and carbon particle (CP) load in each fetal organ. Here we used linear mixed-effects models adjusted for treatment and fetal sex with random intercept effect of dam. All data are expressed as median (Q1; Q3) for the individual statistics (based on average values per fetus) and statistics per litter (based on average values per dam) to show between-fetus and between-dam variability, respectively. *p < 0.05 is considered statistically significant. Abbreviations – CP: carbon particle; DE: diesel exhaust; GD: gestational day

Variable	No. of fetuses	Statistics per fetus Median (Q1; Q3)	Statistics per litter Median (Q1; Q3)	Linear mixed model		
				Estimate	95%CI	*p*-value
Placental CP load						
Fetal weight (g)	56	36 (32; 38)	35 (31; 38)	-4.8	-11; 2.2	0.17
Placental weight (g)	56	7.4 (6.8; 8.4)	8.4 (7.0; 8.8)	0.49	-0.97;1.94	0.50
Placental efficiency	56	4.8 (4.1; 5.4)	4.4 (3.5; 5.2)	-0.73	-1.6; 0.13	0.10
Placenta/fetus weight ratio x 10	56	2.1 (1.9; 2.5)	2.3 (1.9; 2.9)	0.46	-0.021; 0.95	*0.067*
Heart CP load						
Fetal weight (g)	27	36 (32; 38)	35 (29; 36)	-0.60	-8.5; 7.3	0.87
Heart weight (g)	27	0.20 (0.17; 0.24)	0.20 (0.19; 0.23)	-0.023	-0.087; 0.47	0.44
Heart/fetus weight ratio x 10^3^	27	5.7 (5.1; 6.4)	5.9 (5.4; 6.5)	-0.39	-1.9; 0.46	0.67
Kidney CP load						
Fetal weight (g)	27	34 (32; 39)	35 (31; 36)	-3.2	-13; 6.7	0.49
Kidney weight (g)	27	0.17 (0.13; 0.18)	0.15 (0.15; 0.17)	-0.033	-0.11; 0.04	0.34
Kidney/fetus weight ratio x 10^3^	27	4.5 (4.2; 5.0)	4.4 (4.3; 4.7)	-0.47	-1.5; 0.59	0.35
Liver CP load						
Fetal weight (g)	26	34 (31; 38)	34 (32; 39)	0.25	-12; 13	0.97
Liver weight (g)	26	2.4 (1.9; 2.6)	2.4 (1.9; 2.7)	0.039	-1.2; 1.3	0.95
Liver/fetus weight ratio x 10^2^	26	6.8 (6.1; 7.4)	6.8 (5.7; 7.3)	-0.011	-1.6;1.6	0.99
Lung CP load						
Fetal weight (g)	28	35 (31; 38)	35 (32; 38)	1.07	-10;13	0.84
Lung weight (g)	28	0.98 (0.87; 1.1)	1.0 (0.87; 1.2)	0.12	-0.25; 0.48	0.49
Lung/fetus weight ratio x 10^2^	28	2.8 (2.7; 3.0)	2.8 (2.7; 3.0)	0.33	-0.36; 1.0	0.32
Gonad CP load						
Fetal weight (g)	27	36 (32; 38)	35 (29; 39)	4.5	-6.0; 15	0.37

**Fig. 4:**
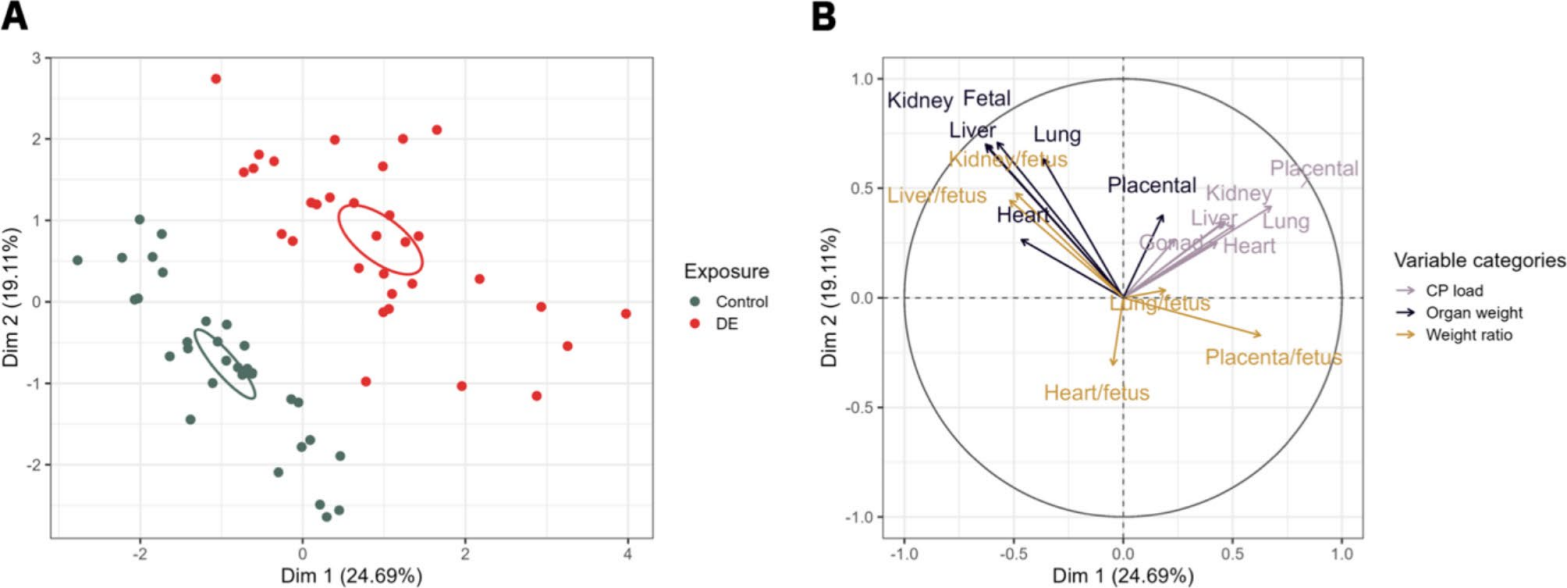
– Plot of individuals (A) and variables factor map (B) of the multiple factor analysis applied on the fetoplacental biometry and CP load profile at GD28. (A) on the plot of individuals, fetoplacental biometric profiles from the DE exposed group (n = 32) are represented by DE in red and fetoplacental biometric profiles from the clean-air exposed group (n = 32) are represented by C in black. The fetoplacental biometric profiles are significantly different between groups, namely between the two exposure groups (i.e., DE exposed and clean-air control) and can be significantly separated along dimension 1 (R^2^ = 0.47, p < 0.01) and dimension 2 (R^2^ = 0.38, p < 0.01). (B) All active variables are plotted into a variable factor map or correlation circle. The purple arrows consider individual organ DE particle loads, the dark blue arrows the placental and fetal weights and the light brown arrows the fetal weight-to-fetal organ weight ratios. The active variables involved in the construction of these dimensions are those whose vectors are the longest and closest to the circle. Abbreviations – CP: carbon particle; DE: diesel exhaust; GD: gestational day

The MFA individual’s factor map (Fig. 4A) shows that a separation of the two exposure groups was possible along dimension 1 (R^2^ = 0.47, p < 0.01) and dimension 2 (R^2^ = 0.38, p < 0.01). The analysis of the qualitative factor shows that the exposed group (DE) is positively correlated to dimension 1 (v.test = 5.4 > 1.96 and cos^2^ = 0.59) and dimension 2 (v.test = 4.9 > 1.96 and cos^2^ = 0.37 far from 1). The separated confidence ellipses show that in both dimensions the group mean is significantly different hence that a specific CP load signature is characterising each group according to their exposure. Dimension 1 and 2 represent 43.8% of the inertia (cumulative percentage of variance) of the data summarised in Table 1. Interpretation of both dimensions is provided by the variable factor map in Fig. 4B. The organ CP loads involved in the construction of these dimension are those whose vectors are the longest and closest to the circle. In this regard, mainly placental CP load is found to positively correlate with dimension 1 (r = 0.68, p < 0.01), but in a lesser degree with dimension 2 (r = 0.42, p < 0.01). In addition, dimension 1 correlates positively with placenta/fetus weight ratio (r = 0.63, p < 0.01) and negatively with fetal weight (r=-0.58, p < 0.01), liver weight (r=-0.63, p < 0.01) and kidney weight (r=-0.63, p < 0.01). In addition, dimension 2 is mostly representing fetal weight (r = 0.71, p < 0.01), kidney weight (r = 0.70, p < 0.01) and liver weight (r = 0.69, p < 0.01). Taking all variables related to fetoplacental biometry and CP load into consideration, we are able to discriminate the diesel exposed from the control group.

**Fig. 5:**
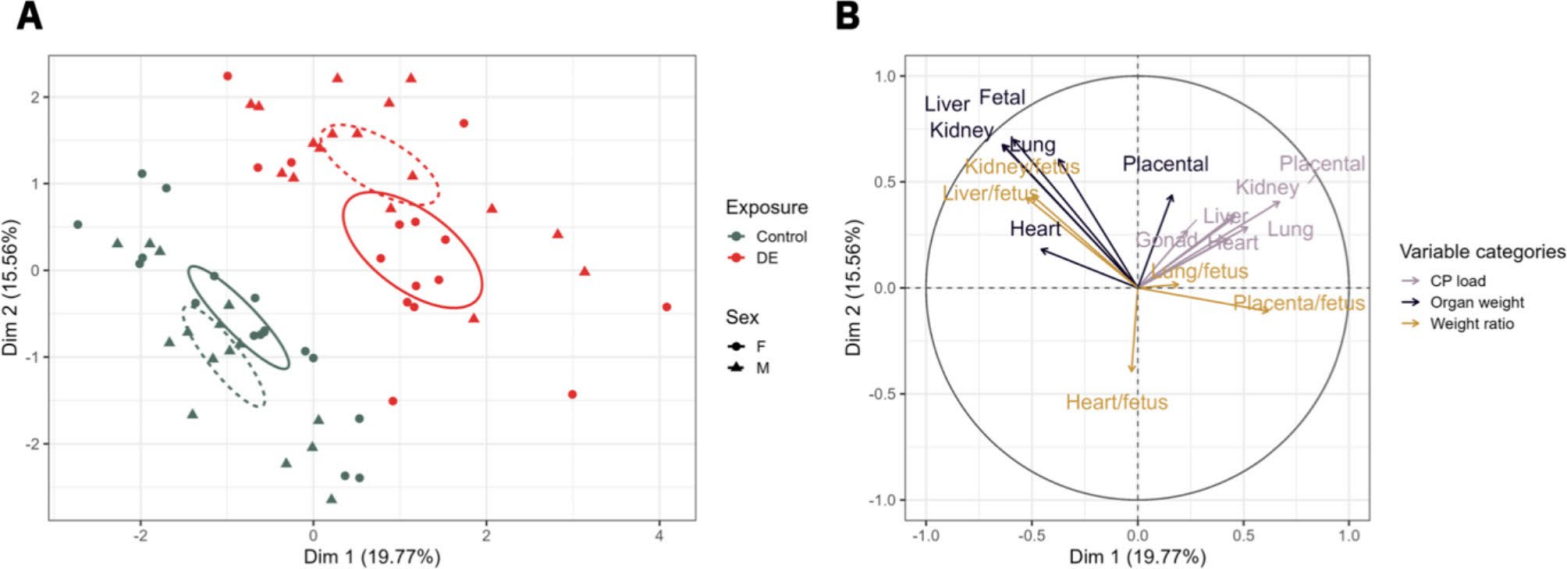
– Plot of individuals (A) and variables factor map (B) of the multiple factor analysis applied on the fetoplacental biometry and carbon particle load profile considering fetal sex at GD28. (A) on the plot of individuals, female (n = 15) and male (n = 17) fetoplacental biometric profiles from the DE exposed group are represented by the solid line and the dotted line in red, respectively. Female (n = 17) and male (n = 15) fetoplacental biometric profiles from the clean-air control group are represented by the solid and dotted line in green, respectively. The fetoplacental biometric profiles are significantly different between groups, namely the two exposure groups (i.e., DE exposed and clean-air control) as well as fetal sex, can be significantly separated along dimension 1 (R^2^ = 0.47, p < 0.01) and dimension 2 (R^2^ = 0.38, p < 0.01). (B) All active variables are plotted into a variable factor map or correlation circle. The purple arrows consider individual organ CP particle loads, the dark blue arrows the placental and fetal weights and the light brown arrows the fetal weight-to-fetal organ weight ratios. The active variables involved in the construction of these dimensions are those whose vectors are the longest and closest to the circle. Abbreviations – CP: carbon particle; DE: diesel exhaust; GD: gestational day

We additionally performed an MFA including fetal sex (Fig. 5A). The profiles differed in a sex-specific way and show that there is a difference in dimension 1 (R^2^ = 0.47, p < 0.01) between both female exposed (DE) and control (C) animals and between male exposed (DE) and control (C) animals. Dimension 2 (R^2^ = 0.46, p < 0.01) explains the difference between male exposed (DE) and control (C) fetuses. The analysis of the qualitative factor shows that the exposed group (both male and female) is positively correlated to dimension 1 (male:v.test = 2.6 > 1.96 and cos^2^ = 0.17; female: v.test = 3.6 > 1.96 and cos^2^ = 0.33) and dimension 2 (only male) (v.test = 4.7 > 1.96 and cos^2^ = 0.42). The separated confidence ellipses show that a specific CP load signature is characterising each group according to their exposure and fetal sex. Dimension 1 and 2 represent 35.4% of the inertia (cumulative percentage of variance) of the data. Interpretation of both dimensions is provided in Fig. 5B. All fetoplacental CP loads are positively correlated with dimension 1, but only placental CP load shows a strong correlation with dimension 1 (r = 0.67, p < 0.01). In addition, dimension 1 correlates positively with placenta/fetus weight ratio (r = 0.62, p < 0.01) and negatively with fetal weight (r=-0.59, p < 0.01), liver weight (r=-0.64, p < 0.01) and kidney weight (r=-0.64, p < 0.01). In addition, dimension 2 is mostly representing fetal weight (r = 0.70, p < 0.01), kidney weight (r = 0.67, p < 0.01) and liver weight (r = 0.68, p < 0.01). Taking all variables related to fetoplacental biometry and CP load into consideration, it is more difficult to discriminate the different groups suggesting an interaction between exposure and fetal sex.

## Discussion

Previously, we have identified the presence of ambient air pollution particles in rabbit (i.e., small particles and CP-like particles indicative of diesel exhaust particles) [8] and human maternal-fetal tissue samples [25, 28] with a particular emphasis on the placenta and its role as a protective barrier for the fetus. In this study, we confirm and elaborate on these findings by identifying the translocation of CPs, originating from diesel engine exhaust, to the fetal circulation and organs in a pregnant rabbit model using our detection technique based on the white-light generation of CPs under ultrashort pulsed laser illumination [25, 26]. Nose-only exposure to diluted diesel engine was conducted from GD3 to GD27 to study and quantify late-stage translocation of combustion-derived particles during gestation in a controlled setting. Increased placental and fetal CP loads were observed following gestational exposure to diesel engine exhaust compared with clean air-exposed controls. In contrast to the previous study by Valentino et al. [8], we are now able to determine a specific CP concentration per sample to highlight a differential accumulation in the various fetal organs. We used our biocompatible and label-free detection technique for detection of CPs from diesel engine exhaust in placental-fetal tissue samples. With this, direct visualisation of the particle distribution in their biological context is possible without the need for sample manipulation. The previous study by Valentino et al. [8] showed no fetal loss during gestation as observed by ultrasound and that the litter size at birth only tended to be lower in the DE exposed group compared to the clean-air exposed group. Moreover, we did not observe a significant difference in terms of the number of fetuses present and alive per litter (median [Q1; Q3]) in the DE exposed group (10 [9; 10]) compared to the control group (8 [7; 9.5]).

In recent years, epidemiological and animal studies have described associations between gestational air pollution exposure and risk for pregnancy, fetal development and neonatal health in later life [8, 9, 23, 29, 30]. Two fundamentally different pathways have been proposed to explain the observed adverse effects on fetal development; a direct and an indirect pathway. Direct developmental toxicity may arise from maternally exposed pollution particles and/or gases that can cross the placental barrier to directly disturb fetal development after reaching the fetal circulation and/or tissues by inducing, among others, inflammation, oxidative stress or epigenetic alterations [16, 31–34]. In this regard, transplacental particle transfer has been previously observed, but the mechanism remains unclear [18, 25, 35–37]. Given the endocytic nature of trophoblastic cells, phagocytosis via fluid-phase endocytosis has been proposed as a possible transport mechanism [36, 38, 39]. On the other hand, the indirect pathway suggests that air pollutants can interfere with fetal development in an indirect manner without being in direct contact with fetal tissues. Maternal- and placental-mediated indirect effects are induced by the accumulation of pollution particles in maternal and placental tissue, respectively, possibly causing organ dysfunction and/or release of secondary mediators (e.g., hormones or cytokines) that could, in turn, interfere with fetal development [40, 41]. The findings presented in this work mainly support the hypothesis of direct effects of prenatal air pollution exposure on fetal development by showing the presence of carbon particles from diesel engine exhaust in fetal tissues. Nevertheless, indirect developmental toxicity due to, for example, intrauterine inflammation, is not ruled out. Additional toxicokinetic studies are paramount to clarify the fate of combustion-derived particles at the maternal-fetal interface.

Regarding the fetoplacental CP distribution, we observed the highest particle load in the placenta compared to the other fetal organs, which is predictable given its barrier function. Moreover, the type of placentation in rabbits is haemodichorial, hence, the maternal blood (by reason containing a higher concentration of CPs compared to the placenta) is in direct contact with the trophoblastic layer. In our previous work [8], we found in the DE exposed group a decrease in the volume fraction of the trophoblastic layer and fetal capillaries but an increase of maternal blood space associated with a decrease of the vascularization index, flow index and vascularization flow index (defined by quantitative power Doppler analysis) in the exposed compared to the control group at GD28. These reduced indices are indicative of a reduction in the fetal vessel density, the blood flow intensity and blood perfusion, respectively, suggesting a decrease in circulating fetal blood in the placenta. The increase in maternal blood space, observed by stereology, and accumulation of CPs along the microvilli of the trophoblastic layer [28] could additionally contribute to the observed high placental CP load in the DE exposed group. Concerning the fetal organs, we observe the highest particle load in the fetal heart followed by the fetal kidneys, whereas a lower number of CPs were detected in the fetal liver and lung. A possible explanation might be the shunting of blood in the fetal circulation to bypass the inactive fetal lungs and liver [42]. Accordingly, the fetal heart receives a large portion of blood, possibly explaining the higher particle load in comparison to the fetal liver and lung, which are bypassed via the ductus venosus and ductus arteriosus, respectively. Nonetheless, it should be taken into consideration that there is no known evidence from literature to support this hypothesis hence future studies are needed to give a more definitive statement on fetal particle distribution.

Taking all variables related to fetoplacental biometry and CP load into consideration, we were also able to discriminate the exposed from the control group as analysed by MFA (Figs. 4 and 5). We observe a good separation along dimension 1 between the exposed (DE) and control (C) groups which is equivalent to (i) an increased CP load and (ii) a decreased fetal organ weight in exposed compared to control pregnant dams. Moreover, we observe an increased placenta/fetus weight ratio in exposed compared to control rabbits. The placenta/fetus weight ratio is a health indicator that reflects whether the relative growth of the placenta and fetus is proportionate and it serves as a common measure of the balance between fetal and placental growth [43, 44]. A high placenta-to-birth weight ratio might indicate a reduced nutrient supply to the fetus [45, 46] and has been associated with long-term cardiovascular disease mortality [47]. The observed effects on fetoplacental biometry might explain the effects previously observed in this experiment, namely that gestationally exposed rabbit fetuses were growth retarded and had their placental function affected [8].

It has been proposed that there is a higher placental vascular resistance in pregnancies with female fetuses compared to pregnancies with male fetuses [48–51]. This permits higher blood flow to male fetuses, which may result in greater particle deposition as observed for metals, more specifically for titanium and silver, in placental tissue and cord blood from male human fetuses [52]. Likewise, persistent organic pollutants were found in higher amounts in male human fetal tissues [53, 54]. Nevertheless, our findings revealed no significant difference in fetoplacental biometry and CP load regarding fetal sex (**Supplementary Table 2** (Additional file 5)) as only a borderline separation was possible between male and female fetuses (Fig. 5A) along dimension 1 after including exposure and fetal sex. However, there might be a potential interaction effect between exposure and fetal sex given the different directions in the exposed (DE) and control (C) group. If no interaction effect would be present, one would see the same trend between male and female fetuses in both the exposed and control group (i.e., female above male in both groups or vice versa).

Despite inhalation exposure being the primary route in environmental and occupational settings, only a limited number of studies examines small sized transplacental particle translocation following inhalation [8, 20, 55–58]. Silver nanoparticles (i.e., 18–20 nm) have been identified within maternal tissues, placentas and fetuses via TEM coupled with energy-dispersive X-ray spectroscopy and single-particle ICP-MS following nose-only inhalation exposure of pregnant female mice to nano-silver aerosols [20]. Similar, in rats, Ti particles were found to accumulate in the placenta and are suggested to reach the fetal tissues following *in utero* exposure to nano-TiO_2_ aerosols but under whole-body exposure during gestation [21]. Contrary to nose-only exposure, as performed in the present study, whole body exposure allows the animals to ingest airborne matter deposited on their fur through licking and/or grooming behaviour, which induces uncertainty on whether inhaled pollution or ingested pollutants are responsible for the observed effects. To note, albeit that the animals were extensively trained to be restrained in the dedicated nose-only tubes, restraining of the animals in the nose-only exposure chamber may increase maternal stress [59, 60], hereby stimulation a sympathetic neural reaction that could have modified the systemic blood flow in rest. This applies to both the DE exposed as the clean-air exposed animals. Moreover, in the present study, rabbits were chosen as animal model over rodents. Humans, rodents and rabbits all share a discoid, haemochorial placenta (i.e., direct contact between trophoblasts and maternal blood). More specifically, haemomonochorial (human at term), haemodichorial (rabbit and human in first trimester) and haemotrichorial (rodent) placentas with one, two and three trophoblastic epithelial layers separating maternal and fetal blood, respectively [61, 62]. Rodents reach their definitive placental structure in a later stage respective to pregnancy length (around 50% of pregnancy in rodents, 38% of pregnancy in rabbits compared to 33% of pregnancy in humans), have less invasive trophoblast cells and have a labyrinthine as opposed to villous organization compared to the human placenta [63–67]. Rabbit placentas also have a labyrinthine structure, yet they are haemodichorial with two trophoblastic layers, which more closely resemble the human placenta [64, 66]. The diesel exhaust exposure (1 mg/m^3^ for 2 h/day, 5 days/week for 20 days over a 31-day gestation, with a mean particle diameter of 69 nm) in the present study equates to a mean exposure of 85 *µ*g/m^3^ over 24 h which is much higher than the 25 *µ*g/m^3^ EU daily limit value for PM_2.5_. However, inhalation exposure was performed during 1 h in the morning and 1 h in the afternoon to mimic the daily commute between home and work [68, 69]. Accordingly, dams were exposed twice a day to a peak concentration rather than being exposed for longer periods to lower concentrations throughout the day and exposure only occurred during gestation without preconceptional exposure hereby complicating the extrapolation to the human situation. Future studies may include sub-groups of various gestational windows to assess the time-dependence of the fetoplacental CP load at each trimester, allowing for the assessments of fetoplacental CP distribution throughout critical gestational exposure windows.

In conclusion, this study confirms our previous findings that maternally inhaled CPs can bypass the placenta to reach the fetal circulation and organs during gestation under current real-life exposure conditions [18, 26] as confirmed here in a controlled exposure setting. The evidence of combustion-derived particle translocation into the fetus is possibly a critical component to explain the observed detrimental effects of prenatal ambient air pollution exposure on fetal development over and beyond the indirect effects (e.g., inflammation and placental dysfunction) caused by particulate accumulation in the maternal lungs.

## Electronic supplementary material

Below is the link to the electronic supplementary material.

Additional file 1

Additional file 2

Additional file 3

Additional file 4

Additional file 5

## Data Availability

The datasets used and/or analysed during the current study are available from the corresponding author on reasonable request.
