# Supplementary material for: Placental-fetal distribution of carbon particles in a pregnant rabbit model after repeated exposure to diluted diesel engine exhaust
Source: Part Fibre Toxicol. 2023 May 18;20:20. doi: 10.1186/s12989-023-00531-z (PMC10193698; doi:10.1186/s12989-023-00531-z)
Supplement: Supplementary file 1 — Additional file 1 [file 12989_2023_531_MOESM1_ESM.docx]

**
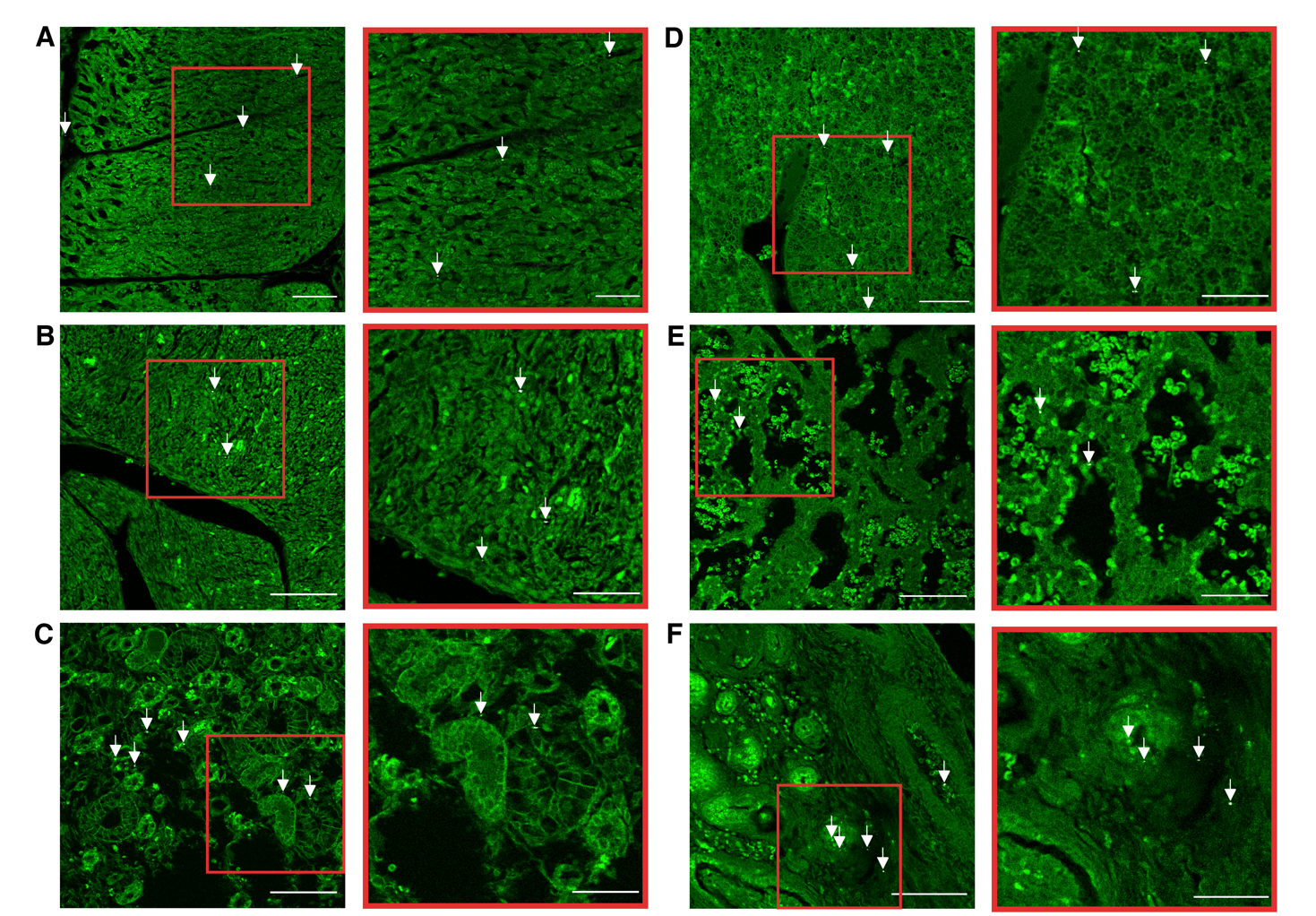
Supplementary Figure 1 – Fetal tissue carbon particle load.** CPs from diesel engine exhaust were present (white and further indicated with arrowhead) in the (A) placenta, fetal (B) heart, (C) kidney, (D) liver, (E) lung and (F) gonads. Scale bar overview images: 100 *µ*m, scale bar zoom-ins: 50 *µ*m. Abbreviations – CP: carbon particle.
