# Supplementary material for: Placental-fetal distribution of carbon particles in a pregnant rabbit model after repeated exposure to diluted diesel engine exhaust
Source: Part Fibre Toxicol. 2023 May 18;20:20. doi: 10.1186/s12989-023-00531-z (PMC10193698; doi:10.1186/s12989-023-00531-z)
Supplement: Supplementary file 2 — Additional file 2 [file 12989_2023_531_MOESM2_ESM.docx]

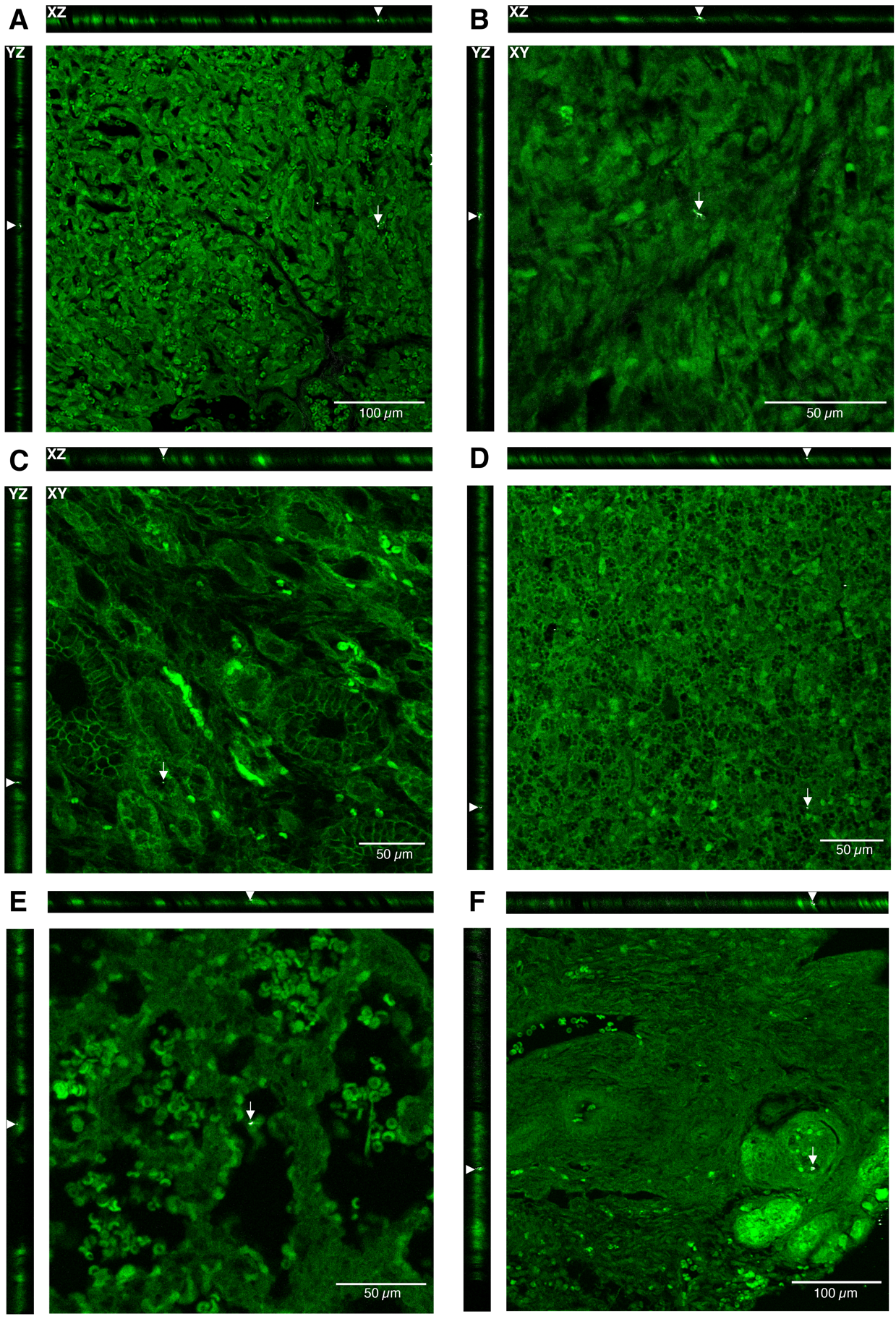
**Supplementary Figure 2 – Fetal tissue embedment of carbon particles from diesel exhaust.** Presence of intra-tissue CPs (white and further indicated with white arrowheads) in the (A) placenta and fetal (B) heart, (C) kidney, (D) liver, (E) lung and (F) gonad. XY-images acquired throughout sections of the respective tissues in the Z-direction and corresponding orthogonal XZ- and YZ-projections showing the embedment of CPs inside the tissue hereby excluding external contamination. Abbreviations – CP: carbon particle.
