# Supplementary material for: Placental-fetal distribution of carbon particles in a pregnant rabbit model after repeated exposure to diluted diesel engine exhaust
Source: Part Fibre Toxicol. 2023 May 18;20:20. doi: 10.1186/s12989-023-00531-z (PMC10193698; doi:10.1186/s12989-023-00531-z)
Supplement: Supplementary file 3 — Additional file 3 [file 12989_2023_531_MOESM3_ESM.docx]

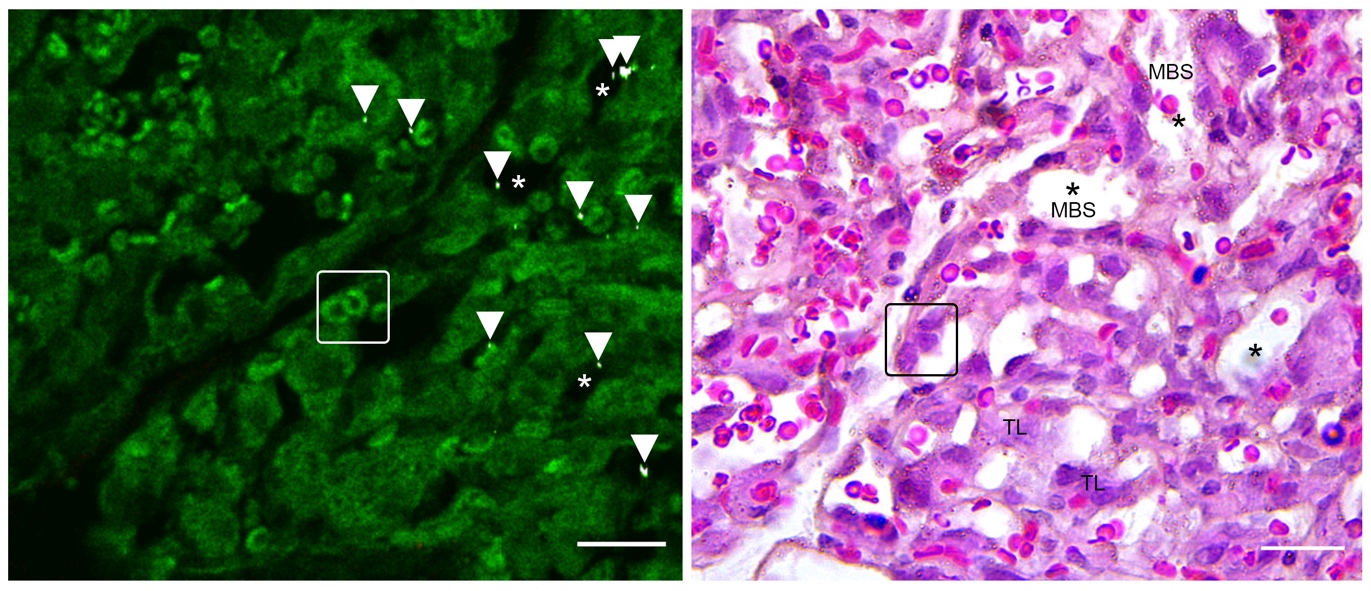
**Supplementary Figure 3 – Localization of carbon particles in the placenta at GD28.** (A) Presence of CPs (white and further indicated with white arrowheads) in the maternal blood space (MBS) of the labyrinthine are in the placenta lumen but also near the trophoblastic layer (TL). (B) Haematoxylin and eosin staining of the serial section. The squared box indicates a group of nuclei to help orientation in both images for better comparison. Scale bars: 20 *µ*m. Abbreviations – CP: carbon particle, GD: gestational day, MBS: maternal blood space, TL: trophoblastic layer.
