# Supplementary material for: Placental-fetal distribution of carbon particles in a pregnant rabbit model after repeated exposure to diluted diesel engine exhaust
Source: Part Fibre Toxicol. 2023 May 18;20:20. doi: 10.1186/s12989-023-00531-z (PMC10193698; doi:10.1186/s12989-023-00531-z)
Supplement: Supplementary file 4 — Additional file 4 [file 12989_2023_531_MOESM4_ESM.docx]

**Supplementary Table 1 –** Diesel exhaust composition during exposure.

|  | **Particle mass concentration (*µ*g/m^3^)** | **Particle number concentration (#/cm^3^)** | **Alveolar surface area (nm^2^/cm^3^)** | **Count median diameter (nm)** | **NO (ppm)** | **NO_x_ (ppm)** | **NO_2_ (ppm)** | **CO (ppm)** |
| --- | --- | --- | --- | --- | --- | --- | --- | --- |
| Mean (SD) | 1014  (133) | 2585000 (249000) | 6420 (1000) | 69  (1) | 25.2 (3.8) | 25.9 (3.6) | 0.7 (0.5) | 5.6 (0.4) |
