# Supplementary material for: Placental-fetal distribution of carbon particles in a pregnant rabbit model after repeated exposure to diluted diesel engine exhaust
Source: Part Fibre Toxicol. 2023 May 18;20:20. doi: 10.1186/s12989-023-00531-z (PMC10193698; doi:10.1186/s12989-023-00531-z)
Supplement: Supplementary file 5 — Additional file 5 [file 12989_2023_531_MOESM5_ESM.docx]

**Supplementary Table 2 – Influence of fetal sex on CP load.** Female pregnant rabbits inhaled either 1 mg/m^3^ of diesel engine exhaust (DE) or clean air (C) for 2h/day, 5 days/week from GD3 to 27. Dams were euthanised and fetoplacental units of control (C) and exposed (DE) groups were collected at GD28. Effect of fetal sex on the placental-fetal CP load was estimated using an unadjusted linear model. All data are expressed as median (Q1; Q3). *p<0.05 is considered statistically significant. Abbreviations – CP: carbon particle.

| Variable | No. of fetuses | Male (%) | Unadjusted linear model | | |
| --- | --- | --- | --- | --- | --- |
|  |  |  | Estimate | CI | *p*-value |
| Placental CP load (#/mm^3^) | 56 | 27 (48.2) | 0.00890 | -0.180; 0.198 | 0.925 |
| Heart CP load (#/mm^3^) | 27 | 13 (48.1) | -0.113 | -0.242; 0.220 | 0.920 |
| Kidney CP load (#/mm^3^) | 27 | 13 (48.1) | 0.0293 | -0.180; 0.239 | 0.776 |
| Liver CP load (#/mm^3^) | 26 | 12 (46.2) | -0.00728 | -0.180; 0.161 | 0.9314 |
| Lung CP load (#/mm^3^) | 28 | 14 (50.0) | -0.148 | -0.305; 0.00835 | 0.0626 |
| Gonad CP load (#/mm^3^) | 27 | 15 (55.6) | -0.0355 | -0.192; 0.121 | 0.645 |
